# Empirical Study of Surface Deterioration Analysis Based on Random Fields for Reinforced Concrete Structures in Marine Environment

**DOI:** 10.3390/ma16114150

**Published:** 2023-06-02

**Authors:** Guixiang Yi, Xinyi Ye, Quanwang Li

**Affiliations:** 1 Central Research Institute of Building and Construction Co., Ltd., MCC, Beijing 100088, China; 2 College of Civil Engineering, Fuzhou University, Fuzhou 350116, China; 3 Department of Civil Engineering, Tsinghua University, Beijing 100084, China

**Keywords:** surface deterioration, concrete structures, durability analysis, spatial variability, empirical study

## Abstract

Corrosion-induced deterioration of the in-service marine reinforced concrete (RC) structures may result in unsatisfactory serviceability or insufficient safety. Surface deterioration analysis based on random fields can provide information regarding the future development of the surface damage of the in-service RC members, but its accuracy needs to be verified in order to broaden its applications in durability assessment. This paper performs an empirical study to verify the accuracy of the surface deterioration analysis based on random fields. The batch-casting effect is considered to establish the “step-shaped” random fields for stochastic parameters in order to better coordinate their actual spatial distributions. Inspection data from a 23-year-old high-pile wharf is obtained and analyzed in this study. The simulation results of the RC panel members’ surface deterioration are compared with the in-situ inspection results with respect to the steel cross-section loss, cracking proportion, maximum crack width, and surface damage grades. It shows that the simulation results coordinate well with the inspection results. On this basis, four maintenance options are established and compared in terms of the total amounts of RC panel members needing restoration and the total economic costs. It provides a comparative tool to aid the owners in selecting the optimal maintenance action given the inspection results, to minimize the lifecycle cost and guarantee the sufficient serviceability and safety of the structures.

## 1. Introduction

For marine reinforced concrete (RC) structures, chloride ions from the outer environment may penetrate through the concrete cover and induce the corrosion of the steel bar, which may result in the surface cracking of the concrete and the loss of the steel’s effective cross-section, leading to the decline of the structure’s serviceability and safety. This durability issue is one of the main concerns of the owners and the users. In current practice, qualitative design is by checking whether the durability parameter values accord with the prescriptive stipulations in codes [1,2,3]; during the service life, periodical or irregular inspections are performed to obtain information regarding their performance states, and the damage extents can be graded according to the qualitative stipulation describing the critical extents of steel corrosion, surface cracking, spalling, etc. in current codes [4].

However, these are not always adequate for the assets’ owners. For the durability design of marine RC structures with long design service life or under extreme environments, the qualitative prescriptive method is not adequate. During the structures’ service, besides the aforementioned assessment of the present state, the prediction and risk assessment regarding the future development of the structure’s surface damage is also essential information. The quantitative approach of surface deterioration analysis is recognized as the advanced tool for the structures in the design and in service to meet the higher demands of the owners. Included in recent codes [3,5,6] and used in practice, it is performed by computing the probability of steel corrosion, which is considered to occur when the chloride concentration on the steel surface reaches the critical level; and according to Fick’s Second Law, the probability can be computed by regarding the durability parameters such as cover depth and apparent diffusion coefficient as random variables.

The current approach to surface deterioration analysis is far from perfect. Due to the spatial variabilities of the durability parameters of materials properties, environmental conditions, and manufacturing quality, the extents of steel corrosion and surface damage of various positions on the same RC members are usually not identical [7,8,9]. As a consequence, the durability analysis based on random variables cannot provide information on the deterioration risk of the RC member [10]. The method of surface deterioration analysis based on random fields was recently developed [11,12,13] and adopted in the recent study on the design and maintenance of marine RC structures [14,15]; it regards the stochastic durability parameters as random fields, adopts several calculation models for various deterioration stages, and by performing the random field simulation, it can be used to assess the “member-level” deterioration risks; e.g., it can provide additional information on the member-level probabilities of surface cracking to various extents at various ages, and maintenance demands can be compared for various design schemes of the newly-built structures.

To better employ the method, the following two problems need to be solved:

(1) The accuracy of the surface deterioration analysis based on random fields is unclear [15]. The verification of the method according to the in-situ inspection data of the existing structures is necessary.
(2) Many studies employed the surface deterioration analysis in newly-built structures [7,10,15], while much fewer studies focused on its application in existing structures, which, in addition, involves the extraction of statistical properties from the inspection data, the interpretation of the grading criteria in the inspection, etc.

This paper presents the empirical study of surface deterioration analysis based on random fields for marine RC structures; inspection data of an in-service high-pile wharf was analyzed in this study. The paper first presents the random field theory and the modeling with the batch-casting effect considered. Then the calculation models for chloride-ingress, crack initiation, and crack propagation of the marine RC structures are introduced. Inspection results of the wharf are presented; statistical analysis of the data is performed to obtain the statistical properties of some key durability parameters. Simulation of the surface deterioration analysis based on random fields for the wharf is performed, and risk assessment results concerning the extent of steel corrosion and surface damage are obtained; quantification of the descriptions on damage extents in inspection is suggested, and damage grading simulation is performed accordingly. Compared to the inspection results, the bias of the surface deterioration analysis based on random fields are shown, and the causes are discussed. Finally, maintenance options given the inspection results are discussed according to the surface deterioration analysis; the number of members requiring restoration and the total cost for each option can be compared to provide additional information for the owners to select from the maintenance options.

## 2. Random Field Modeling

Due to the spatial variability in materials, local environment, and manufactory quality, the durability parameters of marine RC structures exhibit numerical fluctuation spatially [9,16,17]. The random field model is one of the mathematical tools to describe spatial variation [18]. Given a 2-dimensional space, a random field X over Ω is a collection of random variables $\left\{X\left(\omega \right):\omega \in \Omega \right\}$; the mean function $m_{X}\left(\omega \right)$ and the variance function $\sigma_{X}^{2}\left(\omega \right)$ are the collection of the mean values and variance values of the random variables $\left\{X\left(\omega \right):\omega \in \Omega \right\}$; and the function of correlation coefficient $\rho_{X}$, specifying the random field X, quantifies its spatial autocorrelation, with the form of,

(1) $$\rho_{X}\left(\omega_{1},\omega_{2}\right)=\frac{E\left[X\left(\omega_{1}\right)X\left(\omega_{2}\right)\right]-m_{X}\left(\omega_{1}\right)m_{X}\left(\omega_{2}\right)}{\sqrt{\sigma_{X}^{2}\left(\omega_{1}\right)\sigma_{X}^{2}\left(\omega_{2}\right)}}.$$

The random fields of durability parameters are often considered to be discretized for the simplicity of simulation. Under the assumption of isotropy, the autocorrelation coefficient $\rho_{X}\left(\omega_{1},\omega_{2}\right)$ of the discretized random field is the function of the distance between $\omega_{1}$ and $\omega_{2}$; and the following relation is usually adopted in the literature,

(2) $$\rho_{X}\left(\theta \right)=\exp\left(-\frac{\theta^{2}}{\xi^{2}}\right),$$

in which θ is the distance and ξ is the correlation length. By using the Nataf transformation method [19], the 2-dimensional random fields of the durability parameters can be generated according to their autocorrelation coefficient functions.

**Figure 1:**
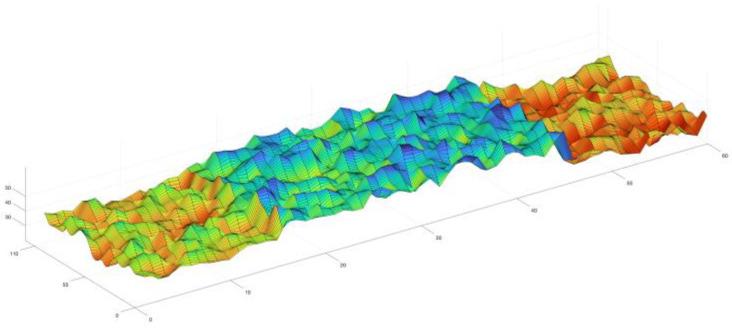
Schematic diagram of “step-shaped” random field.

Batch-casting effects are widely existed in infrastructure constructions and were considered in previous studies [10]; the durability parameter Z is considered as a “step-shaped” random field (as shown in Figure 1 and also in [10]) with the form of,

(3) $$Z=Z_{b}+Z_{a}$$

in which $Z_{b}$ is a random variable representing the variation among batches with the mean of $\mu_{b}$ and the standard deviation of $\sigma_{b}$, and assumed to be independent for different batches, $Z_{a}$ is a normally-distributed random field, modeling the spatial variability within the batch with the mean value of 0 and the standard deviation of $\sigma_{a}$.

Given a collection of inspection data from various batches, the above statistical properties of the step-shaped random field can be estimated by the sample mean and the sample variance:

1. Categorize the data by the batch, and for batch j, the inspection results are denoted as ${\left\{z_{i,j}\right\}}_{i=1,2,\ldots ,n}$; then
2. Calculate the mean value of the data from the same batch, ${\left\{{\bar{z}}_{j}\right\}}_{j=1,2,\ldots ,m}$, which are considered the measures for the random variable $Z_{b}$; then
3. Calculate the sample mean, ${\hat{\mu }}_{b}$, and sample variance, ${\hat{\sigma }}_{b}^{2}$, of the collection ${\left\{{\bar{z}}_{j}\right\}}_{j=1,2,\ldots ,m}$, and regard them as the estimation for $\mu_{b}$ and $\sigma_{b}^{2}$; then
4. Collect the data ${\left\{z_{i,j}-{\bar{z}}_{j}\right\}}_{i=1,2,\ldots ,n;j=1,2,\ldots ,m}$, calculate its sample variance, ${\hat{\sigma }}_{a}^{2}$, and regard it as the estimation for $\sigma_{a}^{2}$.

## 3. Surface Deterioration Models

The ingress of chloride ions may result in the steel depassivation and corrosion in the RC structures. Existing research suggests that there are three phases in the deterioration process, including the chloride-ingress, crack initiation, and crack propagation [20,21].

(1) Chloride-ingress: The chloride ion from the outer environment penetrates into the concrete cover mainly through diffusion, leading to the increase of chloride content in the concrete. When the chloride content around the steel reaches the critical level $C_{\mathrm{cr}}$, it is considered that the corrosion of the steel initiates. Fick’s Second Law is often used to model the process [22], and with the aging effect considered [23], the time duration of this phase can be calculated by Equation (4).
(2) Crack initiation: The corrosion product fills the porous area around the steel and then exerts pressure on the surrounding concrete, leading to surface cracking. Models were developed to predict the time duration of this phase [24,25,26,27], and based on the results from long-term in-situ experiments by [26], Equation (5) was suggested in [10] and adopted in this research.
(3) Crack propagation: The surface crack will propagate in width as the corrosion product on the steel surface is cumulating; as suggested by [28], the transition of the corrosion pattern from pitting corrosion to general corrosion may occur in this phase. The crack width under the pitting corrosion pattern and under the general corrosion pattern can be calculated by Equations (6) and (7), respectively, as suggested by [29,30]; the transition modeling method by [10] is adopted in this study.

The calculation models mentioned above are listed,

(4) $$t_{\mathrm{ini}}=\left\{\begin{array}{cc} {\left[\frac{1}{2D_{a0}t_{0}^{n}}{\left(\frac{x_{0}}{\Phi^{-1}\left(1-\frac{C_{\mathrm{cr}}}{2C_{s}}\right)}\right)}^{2}\right]}^{\frac{1}{1-n}}, & t\leq t_{\mathrm{tr}},\\ \frac{1}{2D_{a0}t_{0}^{n}/t_{\mathrm{tr}}^{n}\;}{\left(\frac{x_{0}}{\Phi^{-1}\left(1-\frac{C_{\mathrm{cr}}}{2C_{s}}\right)}\right)}^{2}, & t>t_{\mathrm{tr}}. \end{array}\right.,$$

(5) $$t_{\mathrm{ci}}=\frac{\Delta A_{s0}}{0.0366d_{b,0}i_{\mathrm{cor},1}},$$

(6) $$w_{p}=0.0575\left(\Delta A_{s}-\Delta A_{s0}\right),$$

(7) $$w_{g}=0.1916\frac{d_{b,0}}{x_{0}}\Delta A_{\mathrm{sm}}+0.164,$$

in which $t_{\mathrm{ini}}$ is the time duration (in seconds) of the chloride-ingress phase, $t_{\mathrm{ci}}$ is the time duration (in seconds) of the crack initiation phase, $w_{p}$ is the crack width (in mm) under the pitting corrosion pattern, $w_{g}$ is the crack width (in mm) under the general corrosion pattern, $x_{0}$ is the cover depth (in mm), $D_{a0}$ is the apparent diffusion coefficient (in 10^−12^ m^2^/s) at the age of $t_{0}$ (in seconds), n is the aging factor, $C_{s}$ is the chloride content on the concrete surface (in the mass percentage of concrete, ${\mathrm{kg}}_{{\mathrm{Cl}}^{-}}/{\mathrm{kg}}_{\mathrm{concrete}}$),$\;C_{\mathrm{cr}}$ is the critical chloride content for steel corrosion initiation (in the mass percentage of concrete, ${\mathrm{kg}}_{{\mathrm{Cl}}^{-}}/{\mathrm{kg}}_{\mathrm{concrete}}$), $t_{\mathrm{tr}}$ is the truncated age (in seconds), $\Phi^{-1}\left(\cdot \right)$ represents the inversed cumulative probability function of standard normal distribution, $d_{b,0}$ is the initial diameter (in mm) of the reinforcement bar, $i_{\mathrm{cor}1}$ is the corrosion current density (in μA/cm^2^) on the surface of the steel that is exposed to chloride ion and before the crack width on the concrete surface reaches 0.3 mm, $\Delta A_{s}$ is the steel cross-section loss (in mm^2^), $\Delta A_{\mathrm{sm}}$ is the average steel cross-section loss between the adjacent stirrups (in mm^2^), and $\Delta A_{s0}$ is the steel cross-section loss (in mm^2^) for concrete surface cracking, which can be calculated by,

(8) $$\Delta A_{s0}=A_{s}\left\{1-{\left[1-\frac{\kappa }{d_{b,0}}\left(7.53+9.32\frac{x_{0}}{\;d_{b,0}}\right)10^{-3}\right]}^{2}\right\},\;$$

where $A_{s}$ is the original cross-section of the steel (in mm^2^), and κ is the factor representing the nonuniform extent of the corrosion, equal to 2 for general corrosion and equal to 4~8 for pitting corrosion.

The steel cross-section loss $\Delta A_{s}$ can be calculated by [10],

(9) $$\Delta A_{s}=\left\{\begin{array}{cc} 0.0366d_{b,0}i_{\mathrm{cor}1}\left(t-t_{\mathrm{ini}}\right), & t\leq t_{0.3};\\ 0.0366d_{b,0}\left[i_{\mathrm{cor}1}\left(t_{0.3}-t_{\mathrm{ini}}\right)+i_{\mathrm{cor}2,0}{\left(t-t_{0.3}\right)}^{0.85}\right], & t>t_{0.3}. \end{array}\right.$$

in which $i_{\mathrm{cor}2,0}$ is the corrosion current density (in μA/cm^2^) on the surface of the steel at $t_{0.3}$, the age (in seconds) when the crack width on the concrete surface reaches 0.3 mm.

By these models, the steel cross-section loss at any age can be calculated, and the width of the crack induced by the corroded steel can be computed accordingly. In addition, combined with the method introduced in Section 2, the stochastic durability parameters can be modeled as random fields to account for their spatial variabilities; and the steel cross-section loss and the crack width are considered time-variant random fields. Therefore, the expansion of steel corrosion and the surface crack propagation in width and in length can be simulated. In this paper, $x_{0}$, $D_{a0}$, $C_{s}$, $C_{\mathrm{cr}}$, $i_{\mathrm{cor}1}$, and $i_{\mathrm{cor}2,0}$ are considered random fields to account for their spatial variability, as shown in Section 4.

Besides the steel corrosion and the surface cracking, the spalling of concrete cover is also typical corrosion-induced damage for marine RC structures. It is common that the spalling of concrete cover is regarded as the consequence of severe cracking. Recent studies [31,32] suggested the essential factors that affect the occurrence probability of spalling, including the crack width, concrete strength, distance between the adjacent steel bars, etc., and empirical models were proposed. However, the high variability of its occurrence is widely recognized [31,33], and currently, it is hard to precisely predict the time, the location, and the area of concrete spalling. According to the mechanical analysis, the cause of its occurrence is the corrosion-induced propagation of surface crack reaching a certain extent and the bonding capacity of the concrete being insufficient to support the gravity of the spalling area. It is reasonable to use a critical state of surface deterioration to represent the occurrence of concrete spalling; e.g., DuraCrete [6] supposed that the spalling occurs when the crack width reaches 1.0 mm; a study by [32] suggested that the critical crack width for longitudinal surface cracking would be much smaller than that for transverse cracking. The aforementioned studies were performed based on theoretical analysis. Based on plenty of inspection data from the Midlands Links motorway viaducts, UK, a study by [34] associated the occurrence of concrete spalling with the steel cross-section loss, and suggested that the cross-section loss reaching 6 mm^2^ is the threshold for the occurrence of concrete spalling. It can be calculated by Equation (6) and the parameters in Section 4.2 that the corresponding critical crack width is about 0.3 mm, which is adopted in this study.

## 4. Empirical Study of Surface Deterioration Analysis

**Figure 2:**
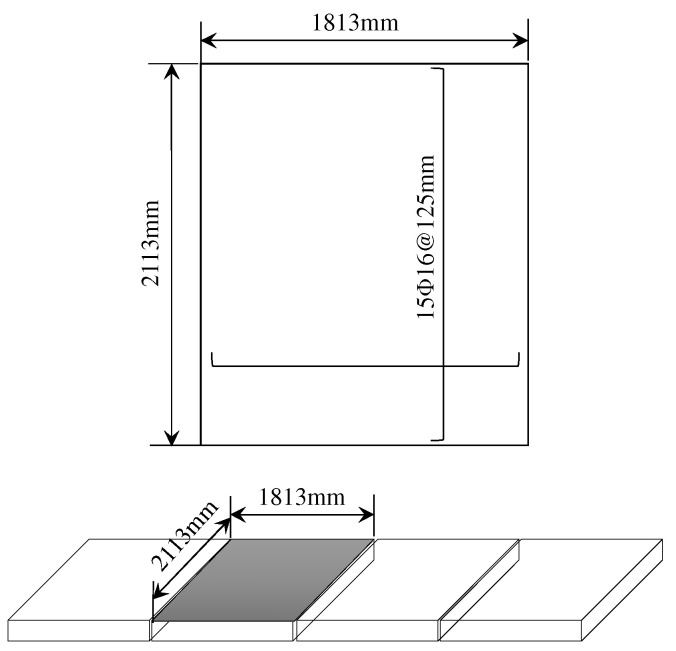
Schematic diagram of the RC panel.

The reinforced concrete panels of an in-service high-pile wharf located northwest of Hong Kong, China, are analyzed in this section. The wharf was built in 1984 and inspected at its service age of 23 years in 2007. The RC panel, with a size of 2113 mm × 7252 mm and a depth of 100 mm, is spliced by four identical precast RC slabs, as shown in Figure 2; in each precast slab, 15 reinforcement bars with a diameter of 16 mm are laid along the long side, and the distances between every two adjacent bars are 125 mm. The characteristic value of the concrete cubic compressive strength of the panel is 30 MPa, and the yield strength of the steel is 360 MPa. There are 144 identical panels on the wharf, and they are exposed in the splashing zone. Since it is the only surface that is exposed to splashing, only the downward surface is analyzed in this section.

**Figure 3:**
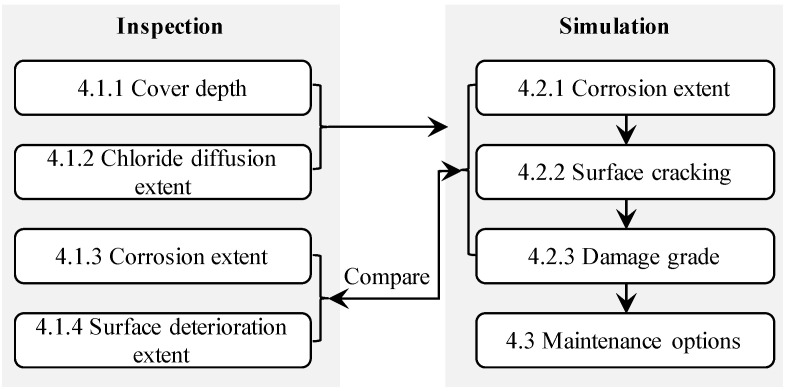
Schematic flow chart for the study in Section 4.

Because the panel consisted of four precast slabs, which are considered to be independently produced, in this section, we consider the batch-casting effect of the panels in terms of the durability parameters, including cover depth $x_{0}$ and apparent diffusion coefficient $D_{a0}$; and the random field modeling method in Section 2 is applied. The flow chart for the study in this section is shown in Figure 3.

### 4.1. Inspection Results

At the age of 23 years, an inspection was performed to gather the information, including the concrete cover depth, the chloride concentration profile of the concrete, steel’s corrosion extent, surface deterioration extent, etc.

#### 4.1.1. Concrete Cover Depth

**Figure 4:**
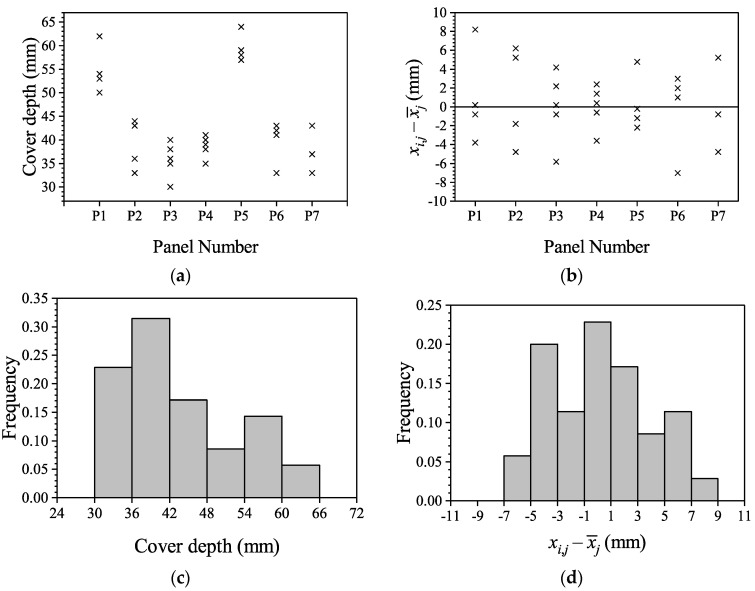
Statistical properties of cover depth: (**a**) Scatters of the cover depths *x*_0_ in various panels; (**b**) Scatters of the “ $x_{i,j}-{\bar{x}}_{j}$” in various panels; (**c**) Distribution of the cover depths *x*_0_; (**d**) Distribution of the “$x_{i,j}-{\bar{x}}_{j}$”.

In the inspection, cover depths were measured for seven panels, randomly selected from the 144 identical panels of the wharf, and denoted as P1, P2, …, P7; five measure points on the same precast slab were randomly selected on each panel, and by using the Electromagnetic-induction Method, the position of outer steel bar can be detected, and the cover depth at the measuring point can be measured. The measured values of cover depths on these seven panels are shown as the scatter plots in Figure 4a; the mean value of the cover depth on each panel can be computed, and the differences between the individual measured value with the mean value on the panel can be calculated, shown as the scatter plots in Figure 4b; histograms of them are plotted, as shown in Figure 4c,d. With the method in Section 2, the statistical properties of cover depth can be estimated accordingly.

#### 4.1.2. Chloride Diffusion Extent

**Figure 5:**
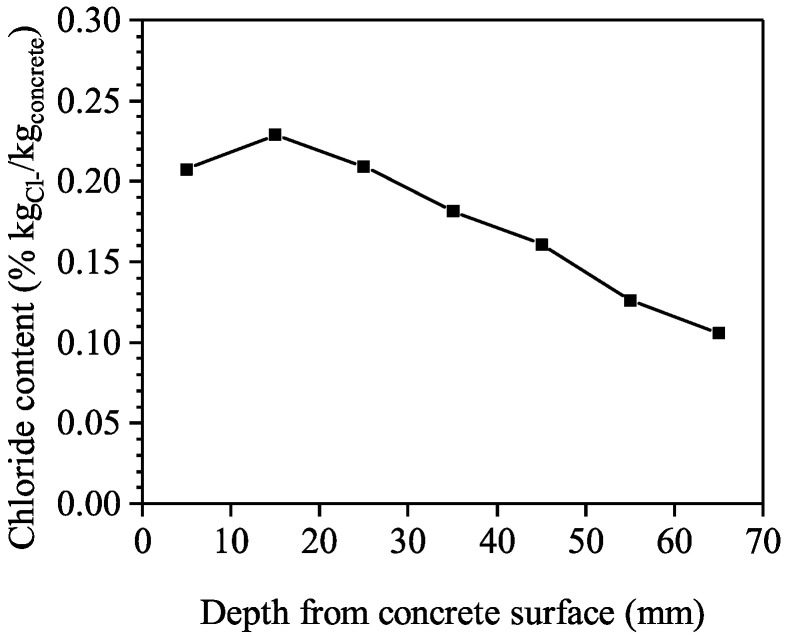
Chloride content profile of the RC slab.

A cylindrical core was extracted on a precast slab, and samples at various depths from the surface were obtained. The chloride content of the sample was measured by the potentiometric titration method. The chloride profile is shown in Figure 5. It can be seen that the peak of the chloride content was measured at a depth of 10~20 mm from the concrete surface; this was reported in existing literature [35,36], where the phenomena were considered due to the following reasons: (1) the border or wall effect during casting; (2) precipitation of brucite; (3) interface between carbonated and noncarbonated layers; (4) interface between the zones of dampening and continuous wetting and drying etc. According to the fitting based on Fick’s Second Law, the surface chloride concentration is 0.27% in ${\mathrm{kg}}_{{\mathrm{Cl}}^{-}}/{\mathrm{kg}}_{\mathrm{concrete}}$ (mass percentage of concrete), and the apparent diffusion coefficient at the age of 23 years is 4.35 in 10^−12^ m^2^/s. The results are considered the mean value of $C_{s}$ and $D_{a0}$ in this study.

**Figure 6:**
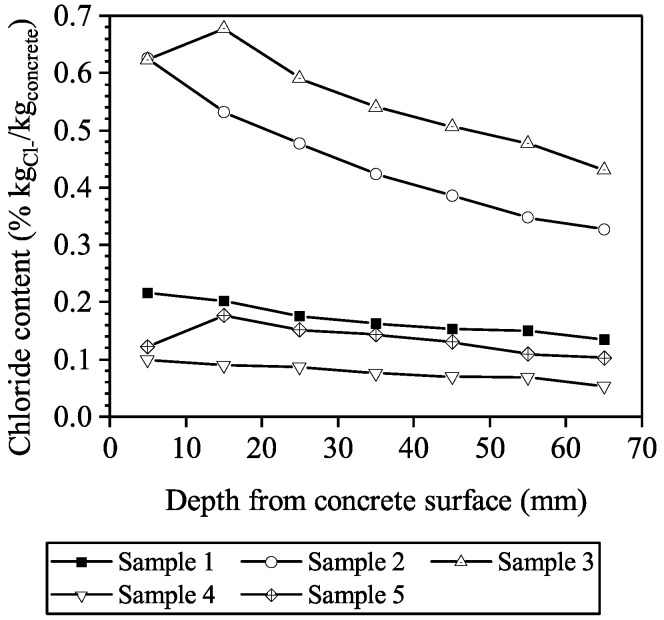
Chloride content profile of the RC beams.

Extractions of a cylindrical core were also made on several surrounding beams with the same concrete mixture and under the same exposure condition; the chloride profiles are shown in Figure 6. It can be calculated that the coefficients of variations of $C_{s}$ is 0.67, and the coefficients of variation of $D_{a0}$ at the age of 23 years is 0.27. Since the surrounding beams were cast with the same concrete mixture and serviced under the same exposure condition as the precast slabs, we assume that the coefficients of variation of $C_{s}$ and $D_{a0}$ of the slabs are the same as those obtained from the inspection results of the beams to consider the variation of the parameters.

#### 4.1.3. Steel Corrosion Extent

Steel corrosion extent was detected by non-destructive testing of half-cell potential on various positions of the RC panels. This method can only provide a qualitative judgment on whether the steel is corroded. In Chinese code [37], those with half-cell potential higher than 350 mV indicate that the steel in those positions is corroded with a confidential level of 90%, those with half-cell potential lower than 200 mV indicate the steel corrosion have not initiated with a confidential level of 90%, and for those with potential between 200 mV and 350 mV, there is no convincing evidence on whether they are corroded or not.

**Figure 7:**
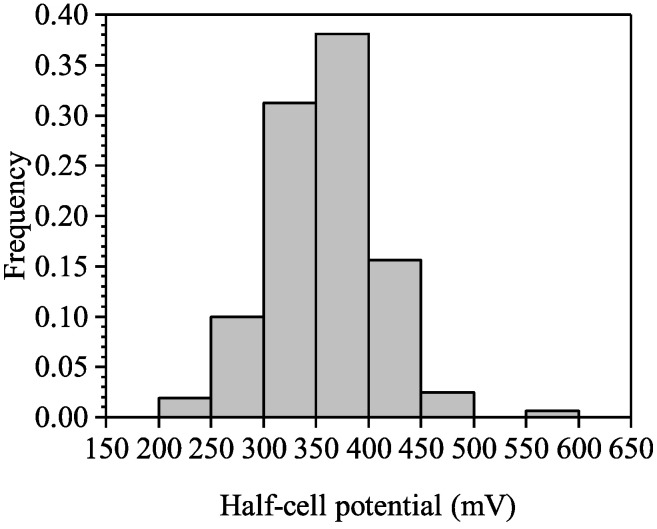
Distribution of half-cell potential.

In this testing, the half-cell potentials were measured at 160 measure points, randomly chosen on the panels, and the testing results of the panels are shown in Figure 7; since there are 54% of the testing results higher than 350 mV, and none is lower than 200 mV, the testing suggests that at least half of the steel bars in panels are corroded.

Steel cross-section loss at the location of concrete spalling on the in-situ panels can also be measured directly. It was found that in some spalling areas, the average steel cross-section loss was up to 194 mm^2^, about 96% of the initial cross-section, and some of them were entirely corroded in cross-section.

#### 4.1.4. Surface Deterioration Extent

The Chinese code [4] classifies the surface deterioration of marine RC structures into four grades, A, B, C, and D, according to the damage extent description regarding steel corrosion, surface cracking, and concrete spalling, as listed in Table 1 is the classification for slab members.

In the inspection in 2007, all the panels of the wharf were inspected, and all the surface damage on the panels was identified. In this project, concrete spalling is the dominant surface damage phenomenon rather than steel corrosion and surface cracking, and the grading was made mainly according to the extent of concrete spalling. Referring to the grading system in Table 1, the inspection agency established the criteria in Table 2 to grade the damage extent.

The above criteria adopted in this inspection are close to, but not exactly, the same as those listed in Table 1. As summarized, among 144 panels, there were 31 panels undeteriorated or slightly deteriorated, regarded as Grade I; there were 54 panels suffering moderate surface deterioration, with less than 1/8 of the surface area damaged, regarded as Grade II; there were seven panels suffering serious surface deterioration, with less than 30% of the surface area was spalled, regarded as Grade III; and there were 52 panels being severely deteriorated regarded as Grade IV, with most of the steel were corroded, and in some locations, the entire cross-section were corroded.

### 4.2. Simulation Results

In this section, random-field simulation is performed to predict the surface deterioration risk of the panels. Models in Section 2 are adopted in this analysis; model uncertainties were measured in [30] and, accordingly, are considered in this section by timing a lognormally distributed random variable with a mean of 1.0 and standard deviation of 0.2. The durability parameters $C_{s}$, $D_{a0}$, $x_{0}$, $i_{\mathrm{cor}1}$, and $i_{\mathrm{cor}2,0}$ are considered as random fields to account for their spatial variabilities, and the parameters n, κ, and $C_{\mathrm{cr}}$ are considered as random variables.

Since the panel consists of four identical precast slabs, in this section, discretized random fields with 15 × 106 elements are established in each precast slab to account for the spatial variability of the durability parameters, and the random field for each parameter in different precast slabs in the same panel is considered to be independent. As introduced in Section 4.1.1, the batch-casting effect is significant for the parameter $x_{0}$, therefore it is considered in this analysis, and the “step-shaped” random field for $x_{0}$ is generated. For the parameters $C_{s}$, $D_{a0}$, $x_{0}$, $i_{\mathrm{cor}1}$, and $i_{\mathrm{cor}2,0}$, there is no sufficient data to quantify the variation between the different precast slabs in the same panel; therefore, it is assumed that the $\sigma_{a}$ for these parameters are equal to 0.1 $\mu_{b}$ according to engineering judgment.

According to the inspection results at the age of 23 years, as introduced in Section 4.1.1 and Section 4.1.2, the statistical properties of $C_{s}$, $D_{a0}$, and $x_{0}$ can be determined. The statistical properties and correlation lengths of $i_{\mathrm{cor}1}$, $i_{\mathrm{cor}2,0}$, $C_{\mathrm{cr}}$, and n are determined according to existing literature [10,17,38]. The statistical properties are listed in Table 3.

#### 4.2.1. Steel Corrosion Extent

**Figure 8:**
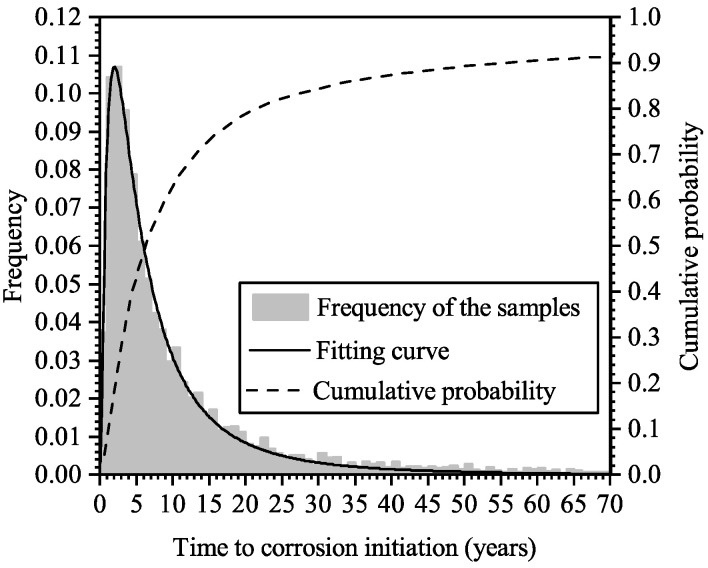
Histogram and cumulative probability of the time to corrosion initiation.

Figure 8 shows the histogram of the simulated time to steel corrosion. In the random-field simulation for an RC panel, if the chloride concentration somewhere on the surface of steel bars reaches the criteria level, $C_{\mathrm{cr}}$, which is the limit concentration level that results in the steel depassivation, the steel bars in the panel are considered as “corrosion initiated”. It can be seen that steel corrosion may initiate very quickly after the construction is completed, and it is most likely to happen around the age of 5 years. The cumulative probability of the time to corrosion initiation is also shown in Figure 8; it can be seen that at the age of 23 years, the probability of corrosion initiated is about 0.8. The results are consistent with the inspection results, which suggested that at the age of 23 years, steel corrosion has been initiated in the majority of the RC panels.

**Figure 9:**
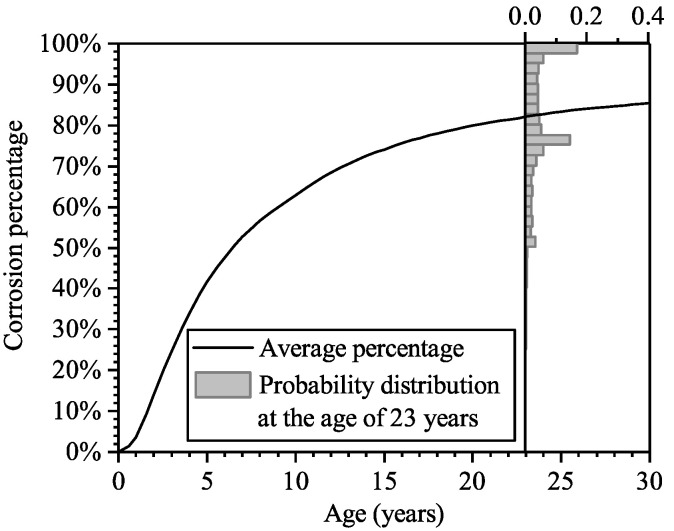
Average corrosion percentage at various ages and the probability distribution of corrosion percentage at the age of 23 years.

The probabilistic distribution of the percentage of corroded steels in one panel is shown in Figure 9. It can be seen that the average percentage at the age of 23 years is around 80%; the histogram of the corrosion percentage exhibits the bimodal characteristic, and the peak values appear at the percentage is about 100% and 75%. As introduced in Section 4.1.3, among the testing results of half-cell potential at random measure points on the panels, more than half of them suggested the steel was corroded with a high confidential level, and the others led to ambiguous judgments on whether it was corroded or not. Simulation results in Figure 9 are consistent with the information from the in-situ inspection, which verifies the accuracy of the random-field simulation.

**Figure 10:**
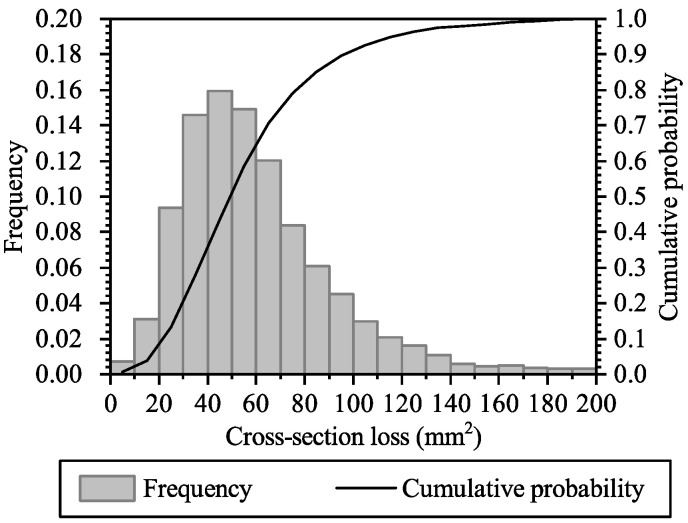
Histogram and cumulative probability of the steel cross-section loss at the age of 23 years.

Figure 10 shows the probability distribution of the maximum steel cross-section loss (in mm^2^) on the RC panel at the age of 23 years. The initial cross-section of the steel is 201 mm^2^; the simulation results suggest that there is a small but unneglectable probability that the entire cross-section of the steel is corroded, and there was such similar observation during the inspection, detailed in Section 4.1.3 and Section 4.1.4. It can be seen from Figure 10 that, at the age of 23 years, the most-likely maximum cross-section loss is about 50 mm^2^, which is about 1/4 of the initial cross-section, and the probability of the maximum cross-section loss exceeding 50 mm^2^ is about 0.5. The results suggest that there may be significant bearing capacity loss on the RC panel at the age of 23 years; the information can be further verified in practice by performing a more advanced and detailed assessment of bearing capacity.

#### 4.2.2. Surface Deterioration

**Figure 11:**
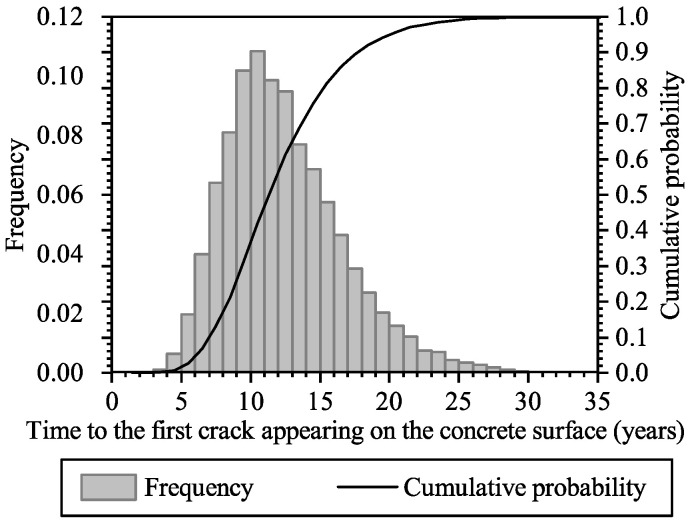
Histogram and cumulative probability of the time to the first crack appearing on the concrete surface.

Figure 11 shows the probability distribution of the time to the first crack appearing on the concrete surface; both the frequency histogram and the cumulative probability curve are shown in the figure. Only the corrosion-induced longitudinal cracks are considered in this analysis. It can be seen that at the age of 5 years, the first crack might appear on the surface of the RC panel, and it is most likely to appear at around 10 years. At the age of 23 years, the probability of at least one surface crack appearing is about 0.95.

**Figure 12:**
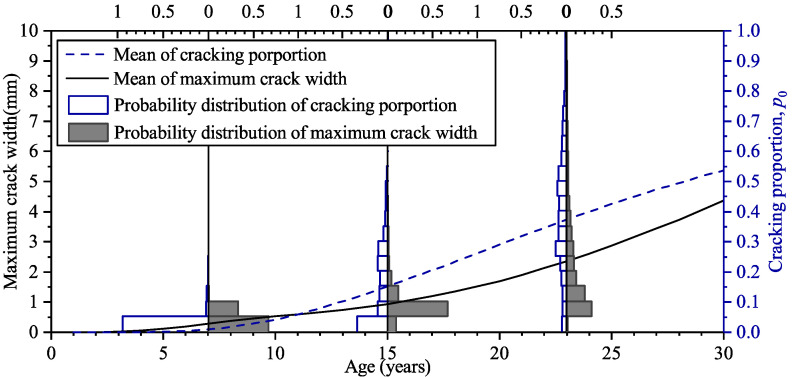
Means of cracking proportion and maximum crack width at various ages and their probability distributions at the age of 7, 15, and 23 years.

Figure 12 shows the increase of the mean of cracking proportion and maximum crack width with time. The probability distribution of cracking proportion and maximum crack width at the ages of 7 years, 15 years, and 23 years are also shown in Figure 12. At the age of 7 years, the probability of the cracking proportion, $p_{0}$, under 0.05 is more than 0.9; after 8 years, the probability decreases to 0.4; and at the age of 23 years, the mean of cracking proportion is about 0.4. The results suggest that the development of surface deterioration of the RC panel is quick; this is because the depth and the material compactness of the cover concrete cannot provide enough barrier for the chloride ingress, and the steel corrosion initiates soon after the exposure to chloride environment, as shown in the previous section.

**Figure 13:**
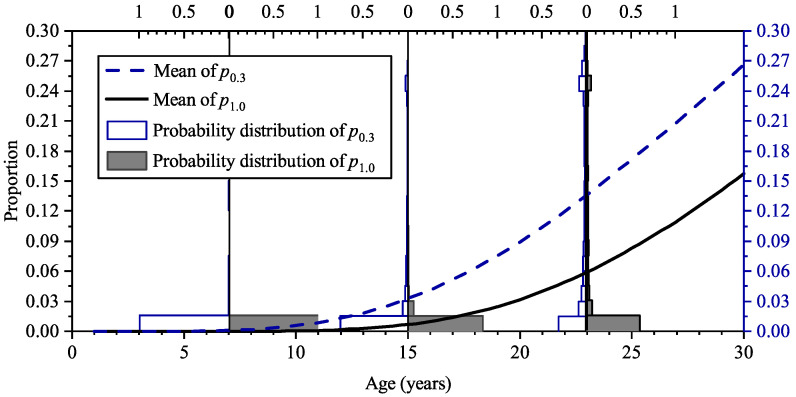
Means of cracking proportion *p*_0.3_ and *p*_1.0_ at various ages and their probability distributions at the age of 7, 15, and 23 years.

Figure 13 shows the increase in the means of cracking proportion with crack width exceeding 0.3 mm and 1.0 mm at various ages; the probability distribution of the cracking proportions at the age of 7 years, 15 years, and 23 years are also shown. In this paper, *p*_0_, *p*_0.3_, and *p*_1.0_ denote the proportion of the cracking surface with the crack width exceeding 0mm, 0.3 mm, and 1.0 mm, respectively. It can be seen that at the age of 23 years, the probability of $p_{1.0}$ exceeding 0.015 is about 0.4, which suggests there is a notable possibility of occurring severe deterioration for the RC panel. It can also be seen that the mean of $p_{0.3}$ and $p_{1.0}$ significantly deviate from the peak values in their probability distribution, which reflects that their probability distributions are long-tailed.

Due to the insufficient recorded data, the inspection results in Section 4.1 does not provide a lot of statistical information regarding surface deterioration. However, the surface deterioration extents are the main issue that affects the damage evaluation in practice, and it is also the basis of performing the damage evaluation simulation in this study, as detailed in the following section; with more data from inspection, these results can be further verified.

#### 4.2.3. Damage Evaluation

As presented in Section 4.1.4, the grading criteria adopted during the inspection are similar to, but not the same as, those suggested in Chinese code [4]. According to the descriptions in Table 2, this study establishes the corresponding quantified grading criteria, as shown in Table 4.

**Figure 14:**
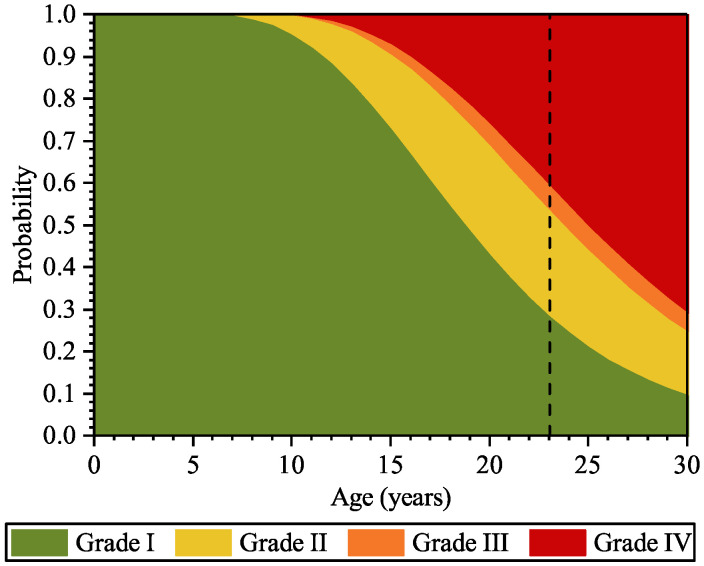
Probabilities of the panel deteriorating to the four damage grades at various ages.

Based on the present system, the damage grading can be simulated. Figure 14 shows the probability of the panels deteriorating to Grade I, Grade II, Grade III, and Grade IV at various ages. It can be seen from the figure that at the age of 23 years, the probabilities of deteriorating to Grade I, Grade II, Grade III, and Grade IV are about 28%, 25%, 6%, and 41%, respectively. A comparison between the simulation results and the inspection results (detailed in Section 4.1) is shown in Table 5. It can be seen that the simulation results coordinate well with the inspection results, which verifies the accuracy of the surface deterioration analysis based on random fields.

Differences between the results in Table 5 may be due to the following reasons:

1. The quantification in Table 2 may not be able to correctly reflect the engineering judgment according to the description of damage extents. For example, as mentioned in Section 4.1, the most frequent surface damage on the panels of this project is concrete spalling instead of cracking; however, the quantified criteria in Table 4 use the indices relating to the crack width to grade the damage extents. It is likely that the quantified criteria are not equivalent to the engineering judgment in the inspection.
2. As shown in Section 4.1, only the mean values and variations of $x_{0}$, $D_{\mathrm{cl}0}$, $C_{s}$ are obtained from the inspection. Because the initial profiles of the project are unavailable, and there is a limitation on conducting more destructive testings on in-service structures, the statistical properties of the other durability parameters are lacking in this study; and they are determined according to the existing literature, which may not be equal to the actual values on this structure. In addition, the model uncertainty may also lead to the bias of the simulation results.

Table 6 shows the comparisons between the in-situ measurements and the simulation results in terms of steel corrosion extent and surface damage grade. It can be concluded from the above analysis that the simulation results are consistent with the in-situ measurements.

### 4.3. Application in the Analysis of Maintenance

In an existing study [10], maintenance strategies with three intervention levels—preventive, necessary, and mandatory—were defined and corresponded to the surface deterioration grades B, C, and D, as listed in Table 7. The same strategies were adopted in this study. Then, we simulate the possible options for the RC panels of this wharf, given the inspection results at its age of 23 years, and compare the maintenance costs.

**Figure 15:**
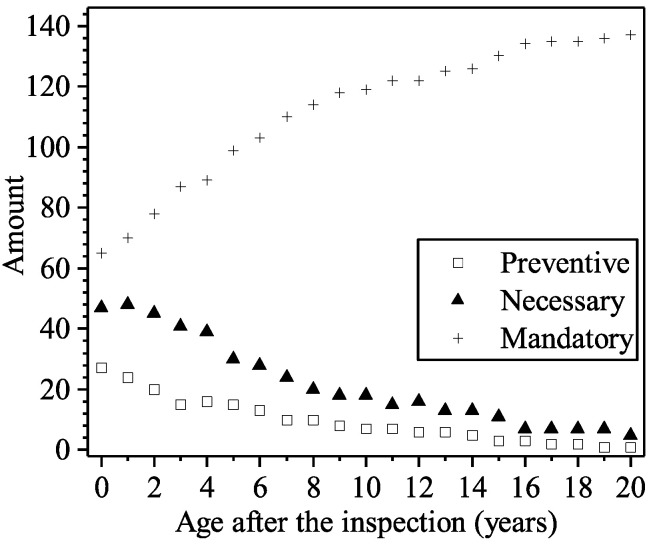
Amounts of panels requiring preventive, necessary, and mandatory maintenance at various ages.

As shown in Figure 15, among the total 144 panels, there are 65 of them in Grade D (mandatory maintenance is required), 27 of them in Grade B (preventive maintenance is required), and 47 of them in Grade C (necessary maintenance is required) at the age of 23 years. If there is no restoration action after the inspection, a failure in safety may occur, or the percentage of the panels in Grade D will increase and eventually approach 100% after 20 years. It can be concluded that postponing the action of maintenance will lead to a more serious deterioration level, which may correspond to a higher-level intervention and a higher unit price for maintenance. However, given that the development of deterioration is a relatively long-term process, and the maintenance is not always urgent, postponing may be financially worthwhile considering the inflation. The owners should balance between these two aspects under the premise of no structural failure.

Given this situation, four options, with the 1st intervention in the present year and the 2nd intervention two years later, are compared in this study. In all four options, the 2nd intervention will restore all the panels in grades B, C, and D; and the damage grades involved in the 1st intervention are different. In this analysis, the differences in the 1^st^ intervention for the four options represent the different decisions of postponing maintenance. Table 8 shows the details of the four maintenance schemes; in the table, the symbol “○” means that the corresponding grade will be involved, and the symbol “-” means that those members in this grade are not included.

The maintenance schemes for the panel members in the three grades are different. For preventive maintenance, quick and low-cost restoring techniques, such as cracking filling, are adopted; for necessary maintenance, medium-cost restoring techniques, such as defective surface removal and patching, are adopted; for mandatory maintenance, high-cost restoring techniques, such as replacement, are adopted. To simplify the discussion, we suppose that those restored panels will not deteriorate again in 2 years, and the restoration will not affect the deterioration process of the unrestored panels.

The total amounts of RC panels being restored for the four options are listed in Table 9. As shown in Table 9, postponing the action of preventive maintenance (adopting Option 2 instead of Option 1) will result in the ongoing deterioration of the panels in Grade B; there will be 11 of them deteriorating to Grade C two years later, and as a consequence, necessary maintenance instead of preventive maintenance will be required for these panels. Postponing the action of necessary maintenance (adopting Option 3 instead of Option 2) will result in the ongoing deterioration of the panels in Grade C; 13 of them will deteriorate to Garde D after 2 years, and mandatory maintenance instead of necessary maintenance will be required.

Fees for the different restoring techniques are assumed according to the engineering practice; fees for preventive, necessary, and mandatory maintenance are assumed as CNY800, CNY 1500, and CNY 5000 per panel, respectively, and the yearly discount rate is assumed as 4%. The total fee (in present value at the structure’s age of 23 years) can be calculated, as shown in Table 9. It can be seen that Option 1 corresponds to the lowest total fee, and Option 3 corresponds to the highest total fee. It should be noted that the ongoing service of the panel member in Grade D without any maintenance may result in structural failure; therefore, although its total fee is lower than the fee of Option 3, Option 4 corresponds to a higher risk of structural failure and a higher potential cost, which is not accounted in this study.

It can be concluded from this study that the random-field-based durability analysis can be used in analyzing the maintenance demands of in-service structures and may provide essential information for comparing and choosing maintenance schemes.

## 5. Limitations and Further Works

The empirical study reported in this paper should be considered in the sight of the following limitations:

1. The random-field-based durability analysis requires the statistical properties of the durability parameters, which should be determined based on a large amount of inspection data; in practice, the data acquisition for all the durability parameters of the in-situ structures is not always convenient.
2. The random-field-based durability analysis relies on the calculation models about the deterioration process. The models used in this study focus on the development of the steel corrosion and surface crack width, which may not be able to evaluate all the deterioration behaviors and phenomena of the marine RC structures. With better models, the accuracy of the durability analysis can be improved.
3. The deterioration behaviors after the restoration cannot be precisely simulated, given the aforementioned methods and models. It is because the restoration often involves using materials that are different from the original, and it may significantly change the mechanism of the structural deterioration process.

In reality, the wharf discussed in this paper was entirely restored in 2008, the next year after the inspection, and is still in service now. Developing the durability analysis method for marine RC structures in their life cycles is one of the future works of the study. In addition, the renewal and update of the models and key parameters are also necessary for improving the accuracy of the random-field-based durability analysis method, which is also one of our future works.

## 6. Conclusions

The presented surface deterioration analysis for reinforced concrete structures serviced in marine environments considers the durability parameters as random fields; by adopting the calculation models for the chloride-ingress, crack initiation, and crack propagation, it is able to predict the risk of surface damage during the structure’s service life. It is verified by the empirical study in this paper, and its application in comparing and choosing the maintenance schemes for the in-service structures is shown. The conclusions can be made as follows.

1. Procedures for extracting the statistical properties of the durability parameters data with the batch-casting effect considered are suggested; it can be used to establish the “step-shaped” random fields in order to coordinate the actual spatial distribution of the parameters.
2. With the statistical properties obtained from inspection results, surface deterioration analysis based on random fields can be performed on the in-service structures; and testing from a high-pile wharf located in the South China Sea was considered as the verification for the analysis;
3. Simulation results of the steel cross-section loss and the crack width were verified in the empirical study in the present paper; surface damage grading was established according to the practical judgment during the inspection of the structure; and it is suggested that the simulation results regarding the simulation results coordinate well with the inspection results;
4. Given the inspection results, by performing the surface deterioration analysis based on random fields, four options for the maintenance schemes were compared in terms of the amounts of panels requiring the restoration and the total economic costs. The results showed that the postponement of intervention would lead to a higher total fee. This information can help to choose alternative options and establish the maintenance scheme for in-service structures.

## Figures and Tables

**Table 1:** Description of the four damage grades in JTJ 302-2006 [4].

Damage Grade	Description of the Phenomenon
Grade A	Without any visible surface deterioration phenomenon.
Grade B	With rust stain, slight corrosion-induced cracks, and concrete hollowing appeared in the small area; the crack widths are less than 0.3 mm.
Grade C	With more rust stain, concrete hollowing, and spalling in the larger area, more corrosion-induced cracks appeared, or net-shaped cracks appeared, with the crack width between 0.3 mm and 1.0 mm; the proportion of concrete spalling in the surface area is less than 30%.
Grade D	With lots of cracks, most or all of the steel corroded, and significant decrease in the steel cross-section occurred; Large area of net-shaped cracks appeared with the crack width exceeding 1.0 mm; Large area of concrete spalling occurred with the proportion in the surface area exceeding 30%.

**Table 2:** Description of the criteria adopted in this inspection.

Damage Grade	Description of the Criteria
Grade I	The steel corrosion is slight, with no surface damaged or a small area of the concrete surface being damaged.
Grade II	Moderate steel corrosion occurred; spalling occurred on less than 10% of the surface area; most of the crack widths are less than 0.3 mm.
Grade III	Serious steel corrosion occurred; Lots of rust stains and corrosion-induced cracks; most of the crack widths are between 0.3 mm and 1.0 mm; the proportion of concrete spalling in the surface area is less than 30%.
Grade IV	With lots of cracks, most or all of the steel corroded, and significant decrease in the steel cross-section happened; Large area of net-shaped cracks appeared with a crack width exceeding 1.0 mm; Large area of concrete spalling occurred with the proportion in the surface area exceeding 30%.

**Table 3:** Statistical properties of durability parameters of the panel.

Parameters	Distribution	Statistical Properties	Correlation Length
*C*_cr_$\;({\mathrm{kg}}_{{\mathrm{Cl}}^{-}}/{\mathrm{kg}}_{\mathrm{concrete}}$)	Lognormal	Mean = 0.10%, std. = 0.02%	2000 mm
*C*_s,b_$({\mathrm{kg}}_{{\mathrm{Cl}}^{-}}/{\mathrm{kg}}_{\mathrm{concrete}}$)	Lognormal	Mean = 0.37%, std. = 0.25%	1960 mm
*C*_s,a_$({\mathrm{kg}}_{{\mathrm{Cl}}^{-}}/{\mathrm{kg}}_{\mathrm{concrete}}$)	Normal	Mean = 0, std. = 0.037%	
*x*_0,b_ (mm)	Lognormal	Mean = 43.0, std. = 8.56	---
*x*_0,a_ (mm)	Normal	Mean = 0, std. = 3.2	130 mm
*D*_a0,b_ (10^−12^m^2^/s)	Lognormal	Mean = 4.35, std. = 1.17	---
*D*_a0,a_ (10^−12^m^2^/s)	Lognormal	Mean = 0, std. = 0.44	250 mm
*n*	Normal	Mean = 0.2, std. = 0.01	---
*κ*	Uniform	Lower bound = 4, upper bound = 8	---
*i*_cor1,b_ (μA/cm^2^)	Lognormal	Mean = 0.67, std. = 0.39	2000 mm
*i*_cor1,a_ (μA/cm^2^)	Normal	Mean = 0, std. = 0.067	2000 mm
*i*_cor2,b_ (μA/cm^2^)	Lognormal	Mean = 8.80, std. = 5.10	2000 mm
*i*_cor2,a_ (μA/cm^2^)	Normal	Mean = 0, std. = 0.88	2000 mm

**Table 4:** Surface damage grading for the panels of the wharf.

Grade	Description of the Criteria	Quantified Criteria
IV	(1) With lots of cracks, most or all of the steel corroded;	(1) *p*_0_ > 50%, and
	(2) Large area of concrete spalling occurred with the proportion in the surface area exceeding 30%;	(2) *p*_0.3_ > 30%, and
	(3) Crack width exceeding 1.0 mm.	(3) *p*_1.0_ > 1%
III	(1) Lots of rust stain and corrosion-induced cracks;	(1) *p*_0_ > 30%, and
	(2) Proportion of concrete spalling in the surface area is less than 30%.	(2) *p*_0.3_ > 10%
II	(1) Most of the crack widths are less than 0.3 mm;	(1) *p*_0_ > 10%, and
	(2) Spalling occurred on less than 10% of the surface area.	(2) *p*_0.3_ > 1%
I	The steel corrosion is slight, with no surface damaged or a small area of the concrete surface was damaged	---

**Table 5:** Comparisons between the simulated results and the actual damage proportions.

Grade	Simulation Results	Inspection Results
I	28%	31/144 = 0.22
II	25%	54/144 = 0.38
III	6%	7/144 = 0.05
IV	41%	52/144 = 0.36

**Table 6:** Comparisons between the in-situ measurements and the simulation.

Items	In-Situ Measurements (At the Age of 23 Years)	Simulation Results
Steel corrosion extent	Half-cell potential test results: 1. <200 mV (not corroded): 0% 2. 200 mV–350 mV (uncertain): 46% 3. >350 mV (corroded): 54%	Figure 8 (probability of corrosion initiation): 1. steel corrosion initiates very quickly after the construction is completed; 2. the probability is about 0.8 at the age of 23 years. Figure 9 (corrosion percentage in each panel at the age of 23 years): 1. the average percentage is about 0.8; 2. the probability of average percentage around 0.75 is significant.
	Visual inspection in spalling area: 1. average cross-section loss of the exposed steel was up to 194 mm^2^; 2. some were entirely corroded in cross-section.	Figure 10 (probability of steel cross-section loss at the age of 23 years): 1. the most-likely maximum cross-section loss is 50 mm^2^; 2. the probability of the entire cross-section of the steel being corroded is small but unneglectable.
Surface damage grade	Percentage of panels in various damage grades: 1. Grade I: 31/144 = 0.22; 2. Grade II: 54/144 = 0.38; 3. Grade III: 7/144 = 0.05; 4. Grade IV: 52/144 = 0.36.	Figure 14 and Table 5 (Percentage of panels in various damage grades): At the age of 23 years, 1. Grade I: 28%; 2. Grade II: 25%; 3. Grade III: 6%; 4. Grade IV: 41%.

**Table 7:** Durability damage grade for concrete structures [10].

Grade	Item in JTJ-302	Our Quantification
A	Without any visible cracking	*p*_0_ ≤ 1%
B	A few corrosion-induced cracks with width less than 0.3 mm	*p*_0_ > 1% and *p*_0.3_ ≤ 1%
C	Some continuous cracks along the reinforcement bar, with width between 0.3 mm and 3.0 mm	*p*_0.3_ > 1% and *p*_3.0_ ≤ 1%
D	A large number of continuous cracks along the reinforcement bar, with some crack widths exceeding 3.0 mm	*p*_3.0_ > 1%

**Table 8:** Maintenance schemes with four options.

Maintenance Schemes		Grade B	Grade C	Grade D
Option 1	1st intervention	○	○	○
	2nd intervention	○	○	○
Option 2	1st intervention	-	○	○
	2nd intervention	○	○	○
Option 3	1st intervention	-	-	○
	2nd intervention	○	○	○
Option 4	1st intervention	-	-	-
	2nd intervention	○	○	○

**Table 9:** Total fees and the amounts of the panels requiring restoration for the four options.

Maintenance Schemes		Grade B	Grade C	Grade D
Option 1	1st intervention	27	47	65
	2nd intervention	4	0	0
	Total amount	31	47	65
	Total fee	CNY 420,059		
Option 2	1st intervention	0	47	65
	2nd intervention	20	11	0
	Total amount	20	58	65
	Total fee	CNY 425,548		
Option 3	1st intervention	0	0	65
	2nd intervention	20	45	13
	Total amount	20	45	78
	Total fee	CNY 462,297		
Option 4	1st intervention	0	0	0
	2nd intervention	20	45	78
	Total amount	20	45	78
	Total fee	CNY 437,777		

## Data Availability

Data is available in a publicly accessible repository that does not issue DOIs.
